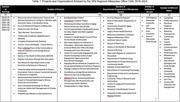# Fostering Bi‐directional ADRD Health Equity Research: Insights from the Wisconsin Alzheimer's Institute Regional Milwaukee Office Community Advisory Board

**DOI:** 10.1002/alz70860_105991

**Published:** 2025-12-23

**Authors:** Gina Green‐Harris, Stephanie Houston, Celena M Ramsey, Maria C Mora Pinzon, Tamara J. LeCaire, Gail D. Morgan, Darona Woods, Jennifer Landeta‐Vidal, Nia C Norris

**Affiliations:** ^1^ Wisconsin Alzheimer's Institute Regional Milwaukee Office, University of Wisconsin School of Medicine and Public Health, Milwaukee, WI, USA; ^2^ Center for Community Engagement and Health Partnerships, Milwaukee, WI, USA; ^3^ UW‐ Wisconsin Alzheimer's Institute, Milwaukee, WI, USA; ^4^ Wisconsin Alzheimer's Institute Regional Milwaukee Office, University of Wisconsin‐Madison School of Medicine and Public Health, Milwaukee, WI, USA; ^5^ Department of Medicine, Division of Geriatrics, School of Medicine and Public Health, University of Wisconsin‐Madison, Madison, WI, USA; ^6^ Wisconsin Alzheimer's Institute, University of Wisconsin School of Medicine and Public Health, Madison, WI, USA; ^7^ Wisconsin Alzheimer's Institute Regional Milwaukee Office‐ University of Wisconsin School of Medicine and Public Health, Milwaukee, WI, USA; ^8^ Wisconsin Alzheimer's Institute, University of Wisconsin School of Medicine and Public Health, Madison, WI, USA

## Abstract

**Background:**

To address the heightened need to improve health equity in Alzheimer's disease and related dementias (ADRD), participatory research activities in partnership with African American community members must be promoted. The Wisconsin Alzheimer's Institute (WAI) Regional Milwaukee Office established a Community Advisory Board (CAB) to foster academic‐community partnerships, advise investigators on research design and strategies to advance African American research programs and increase participation, and improve primary, secondary and tertiary prevention for ADRD health disparities. We sought to understand the CAB's perceptions on research priorities and their value to investigators, to promote CABs in ADRD‐related health equity research.

**Method:**

Descriptive analysis of CAB performance and activities were provided. Data was collected from meeting notes of CAB meetings held 6‐8 times annually during 2019‐2024. CAB members participated in 2 strategic planning meetings and completed a satisfaction survey in November 2022.

**Result:**

The CAB is intergenerational and ethnically diverse; 97% African American and 3% white and Native American, with 20% men and 80% women. Members include caregivers, research participants, people living with dementia, and well‐known influential community members in the African American community knowledgeable about ADRD and active in volunteering. Service in the CAB ranges from a few years to over 15. For 27 different projects, the CAB provided advice on study design, recruitment, retention, and community engagement strategies across 17 different research areas including caregiving, family dynamics, driving safety for older adults, communicative disorders, diabetes, and lifestyle education (Table 1). Advice included culturally tailored outreach strategies, recruitment approaches, relationship building, transparency, and resources for community‐based efforts. CAB members described that the benefits of participating in the CAB include increased awareness, knowledge, resources about ADRD, and having a platform to amplify voices and positively impact community‐based participatory research. Future directions for the CAB include action planning to prioritize ADRD and health equity research while promoting evidence‐based interventions.

**Conclusion:**

The WAI Regional Milwaukee Office CAB model supports ADRD and health equity research by enhancing CAB members’ knowledge, fostering connections between investigators and the community, and advising on study designs and research activities to increase African American participation in ADRD health equity research.